# Trauma-Induced Coagulopathy in Rat Models: Assessing Hemostatic Changes in Mild and Severe Traumatic Brain Injuries

**DOI:** 10.3390/neurolint18040073

**Published:** 2026-04-20

**Authors:** Refat Aboghazleh, Shrouq Al-Sabaileh, Mustafa Nadi, Walid Aburayyan, Mohammad Saadaldin, Ahiam Awadat, Mohammad Badawi, Mimas Al-Helalat, Afnan Atiyat, Manal Udwan, Abdel Latif Al-Houwari, Abdulraheem Alhourani, Abdalraman Al-eyadah, Radwan Sabayleh, Nesrin Seder

**Affiliations:** 1 Department of Basic Medical Sciences, Faculty of Medicine, Al-Balqa Applied University, Al-Salt 19117, Jordan; 2 Department of Pathology and Forensic Medicine, Faculty of Medicine, Al-Balqa Applied University, Al-Salt 19117, Jordan; 3 Department of Special Surgery, Faculty of Medicine, Al-Balqa Applied University, Al-Salt 19117, Jordan; 4 Department of Medical Laboratory Sciences, Faculty of Science, Al-Balqa Applied University, Al-Salt 19117, Jordan; 5 Faculty of Medicine, Al-Balqa Applied University, Al-Salt 19117, Jordan; 6 Faculty of Medicine, Cairo University, Cairo 12613, Egypt; 7 Department of Pharmaceutical Chemistry and Pharmacognosy, Faculty of Pharmacy, Applied Science Private University, Amman 11937, Jordan

**Keywords:** traumatic brain injury, coagulopathy, D-dimer, fibrinogen, fibrinolytic parameters

## Abstract

***Background****:* Traumatic brain injury (TBI) has been associated with coagulation disorders, and coagulation and fibrinolytic parameters are frequently monitored in the acute stage of TBI. ***Methods****:* Using a rat closed head injury model, mild and severe TBIs were induced. Blood samples were obtained at five post-injury time points, including 1 day and 1, 2, 3, and 4 weeks, to assess coagulation and fibrinolytic parameters, specifically prothrombin time (PT), partial thromboplastin time (PTT), D-dimer, and fibrinogen. ***Results****:* In mild TBI, all hemostatic parameters remained largely within physiological ranges, despite minor statistical fluctuations in PT and PTT. Conversely, severe TBI resulted in significant elevations of PT (*p* = 0.00015) and PTT (*p* = 0.01) during the first week. Additionally, D-dimer levels increased significantly at week 2 (*p* = 0.024) and week 4 (*p* = 0.014) post-injury, surpassing the upper limit of normal. Although fibrinogen levels showed a significant increase at week 2 compared to the control group (*p* = 0.011), they remained within the normal reference range. ***Conclusions****:* While mild TBI is characterized by stable hemostatic markers, severe TBI demonstrates a clear and significant progression from acute coagulation activation to secondary fibrinolysis. These findings suggest that severe TBI-induced coagulopathy is a progressive event requiring extended longitudinal monitoring beyond the initial acute phase.

## 1. Introduction

Traumatic brain injury (TBI) and its severity have garnered significant attention not only due to the acute damage and immediate consequences but also because of the long-term impact on patients’ overall well-being and quality of life [1,2]. Common neurodegenerative diseases, psychological disorders, and hemostatic complications associated with TBI include epilepsy [3,4], cognitive impairment [5,6], Alzheimer’s disease [7,8], depression [9,10], stroke [11], intracranial hemorrhage [12,13], and disruption of the blood–brain barrier (BBB) [14,15,16], often accompanied by blood leakage.

TBI has been associated with disordered coagulation [17,18,19]. Following TBI, hypercoagulability and hyperactivation of fibrinolytic pathways were reported in impacted brains in response to vascular endothelial cell damage [17,18,20]. Coagulation and fibrinolytic parameters undergo dynamic changes in response to these pathologies. Following injury, fibrinogen is consumed and broken down, with its levels reaching their lowest point between 3 and 6 h post-injury. Hypercoagulability triggers heightened fibrinolytic activity, leading to a rapid increase in plasma D-dimer levels, which peak around 3 h after TBI [20,21]. Hyperactivation of the fibrinolytic pathway exacerbates bleeding, which is one of the most critical complications following TBI [13,22,23]. In addition, the interplay between fibrinolysis and consumptive coagulopathy following TBI elevates the risk of delayed or worsening intracranial hemorrhage [24,25].

For a long time, it has been established that TBI results in disruption of the BBB, microvascular injury and leakage, and impaired neurovascular coupling [14,15,16,26,27,28,29,30].

The coagulopathy is usually defined by the coagulation parameters, including prothrombin time (PT), partial thromboplastin time (PTT), D-dimer, and fibrinogen [12,20,22,31,32,33].

The changes in these parameters were seldom examined in relation to the severity of the head injury and the specific time points at which they were measured and recorded.

In a closed head injury rat model, transient microthrombi were observed within 1 h following mild TBI [11]. Elevated levels of D-dimer have been reported in patients with aneurysmal subarachnoid hemorrhage [21,34] and stroke [35], indicating activation of systemic coagulation. Additionally, elevated D-dimer levels in patients may serve as a predictor of poor prognosis and an increased risk of progressive hemorrhagic injury [12,36]. Changes in D-dimer levels can occur rapidly and be detected in the blood within minutes following the TBI [37].

The fibrinolytic (hemorrhagic) phenotype of disseminated intravascular coagulation (DIC) has been observed in the early phase of TBI, as indicated by elevated D-dimer and fibrin/fibrinogen degradation products (FDP) [23], and is associated with poor outcomes. Additionally, an elevated D-dimer/fibrinogen ratio has been shown to predict progressive hemorrhagic injury following TBI [12].

Hemostatic parameters, including PT, PTT, and platelet count, were measured in patients with isolated TBI, revealing a 54% increase in these values within the first 24 h post-injury [31].

The risk of intracranial hemorrhage (ICH) progression was found to be five times higher within the first 24 h post-TBI in patients with abnormal PTT [38], with an associated increase in mortality rates, particularly in severe TBI cases.

In this study, we investigated hemostatic changes following mild and severe TBI over a 4-week period, divided into five intervals: 1 day, 1 week, 2 weeks, 3 weeks, and 4 weeks post-injury. This investigation aims to expand our understanding of the hemodynamic alterations associated with different severities of TBI, potentially guiding treatment strategies over the 4-week period following head injury.

## 2. Methods

All procedures were performed following institutionally approved protocols in accordance with the Institutional Review Board Protocol No. 1440/1/3/26 at Al-Balqa Applied University.

A total of 200 adult male Sprague Dawley rats (8–10 weeks old, 225–250 g) were randomly assigned to 20 subgroups (*n* = 10 per group). Half of the animals (10 subgroups, *n* = 100) were allocated to mild TBI and control groups, while the other half (10 subgroups, *n* = 100) were assigned to severe TBI and control groups. Each set of 10 subgroups was further divided into 5 subgroups for TBI and 5 subgroups for controls, based on five post-injury time points (1 day and 1, 2, 3, and 4 weeks). Following TBI, animals were maintained for the specified intervals before being sacrificed. The animals were housed in pairs per cage and had ad libitum access to food and water. They were maintained under a standard 12:12-h light–dark cycle. They were obtained from the animal facility at Jordan University of Science and Technology. All experiments were conducted between 10:00 AM and 3:00 PM.

To minimize bias and ensure the validity of our results, animals were randomly assigned to experimental groups by an independent researcher who was not involved in the experimental procedures or data analysis. Each rat was assigned a coded number that did not reveal its group identity, and during the experimentation phase, the experimenter analyzing the coagulation and fibrinolytic parameters was blinded to the group assignments. Statistical analysis was performed by a separate researcher who received datasets without group identifiers, ensuring that the analysis was conducted without knowledge of the group assignments. Additionally, outcome assessments were conducted in a blinded manner by a third-party evaluator who was unaware of the experimental conditions and group assignments.

### 2.1. TBI Induction

**Figure 1:**
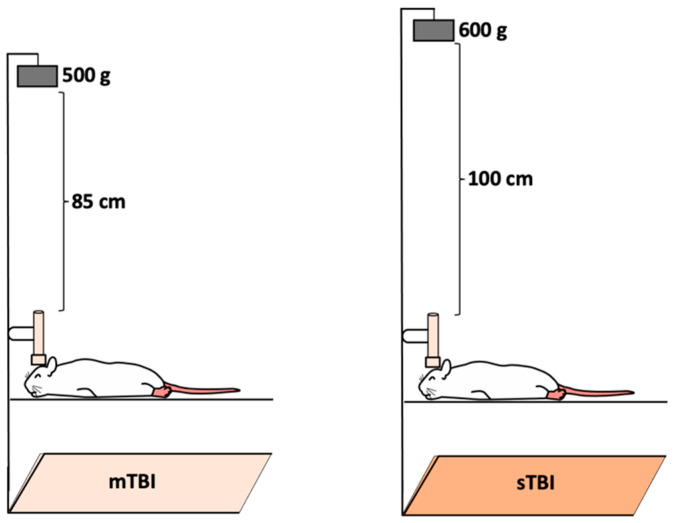
Schematic illustration of the weight-drop closed-head injury model used to induce mild and severe traumatic brain injury. mTBI: mild TBI, sTBI: severe TBI.

A modified weight-drop closed head injury model [15,16,39,40] was employed to induce mild and severe TBI. Rats were sedated using intraperitoneal injections of ketamine (80 mg/kg) and xylazine (10 mg/kg), with the assessment of toe-pinch response every 2 min until absent. The rats were placed in a prone position on a flat aluminum foil (Figure 1) secured to the top of a plexiglass box (40 × 20 × 20 cm). A blunt tip of a metal pin (1 cm in diameter, 12 cm in length) was positioned on each rat’s head above the parietal bones along the midline. Ketamine and xylazine were used in this study based on their effectiveness in providing deep and stable anesthesia suitable for the experimental procedures. For mild TBI, a 500 g weight was freely dropped from a height of 85 cm along a metal guide rail, inducing a single hit. To induce severe TBI, the weight was increased to 600 g and the height to 100 cm, resulting in a mortality rate of 5% (5 out of 100 animals). Mortality was recorded throughout the study period. Deaths occurred only in the severe TBI group during the first 24 h of the post-injury phase. Control groups underwent the same anesthesia protocol but were not subjected to head injury.

The TBI model used in this study has been previously developed and validated in our laboratory. Injury severity was characterized in earlier studies using a combination of neurobehavioral, imaging, and pathological criteria [15,16]. Mild TBI was defined by a transient reduction in neurological score at 10 min post-impact, low mortality, and absence of intracranial bleeding or gross structural injury, as assessed by MRI and post-mortem examination. Neurobehavioral assessment included open field, beam walk, and inverted wire mesh tests. In contrast, severe TBI in this model has been associated with higher mortality rates, reaching up to 20% in previous studies [15,16].

### 2.2. Blood Sampling

The animals were deeply anesthetized and humanely euthanized in accordance with the guidelines set by the Institutional Review Board, ensuring alignment with ethical and scientific standards. After confirming complete anesthesia, the chest was opened, and a blood sample was withdrawn directly from the left ventricle of the heart. The animals were allowed to die by exsanguination.

Blood samples were collected from the heart at specific time intervals following TBI, including 1 day and 1, 2, 3, and 4 weeks post-injury. A total of 195 plasma samples (100 from the mild TBI group and 95 from the severe TBI group) were directly extracted and stored at −40 °C until further use. The normal reference ranges for coagulation parameters were defined based on rat-specific standards provided by the manufacturer for the ELISA kits (D-dimer and fibrinogen) and previously published rat studies for PT and PTT. The reference ranges used in this study were as follows: PT (13.9–21.1 s) [41], PTT (15.2–33.9 s) [41], D-dimer (3–200 ng/mL), and fibrinogen (10–800 ng/mL).

### 2.3. Measurement of Fibrinogen and D-Dimer

The plasma levels of D-dimer and fibrinogen were measured by a quantitative sandwich enzyme immunoassay technique (ELISA) using commercial kits (Sunlong Biotech, Hangzhou, China).

#### 2.3.1. Fibrinogen (Fbg) Test

Total fibrinogen (Fbg) levels were determined using a rat Fbg ELISA kit (Sunlong Biotech, SL0280Ra, China). The micro-ELISA strip plate included in the kit was pre-coated with an antibody specific to Fbg. Standards and samples were added to the appropriate wells and bound to the specific antibody. Subsequently, a horseradish peroxidase (HRP)-conjugated antibody specific for Fbg was added to each well, followed by incubation. After washing to remove unbound components, a TMB substrate solution was added. Wells containing Fbg and the HRP-conjugated Fbg antibody turned blue and changed to yellow upon addition of the stop solution. The optical density (OD) was measured spectrophotometrically at a wavelength of 450 nm, with OD values proportional to the Fbg concentration. The Fbg concentrations in the samples were calculated by comparing the sample OD values to the standard curve.

#### 2.3.2. D-Dimer Test

D-Dimer (D2D) levels were measured using a rat D2D ELISA kit (Sunlong Biotech, SL1303Ra, China). The micro-ELISA strip plate provided in the kit was pre-coated with an antibody specific to D2D. Standards and samples were added to the designated wells, where they bound to the specific antibody. Following this, an HRP-conjugated antibody specific to D2D was added to each well and incubated. Unbound components were washed away, and a TMB substrate solution was introduced. Wells containing D2D and the HRP-conjugated D2D antibody turned blue and then yellow after the stop solution was added. The OD was measured at 450 nm, with the OD value being proportional to the D2D concentration. The concentrations of D2D in the samples were calculated by comparing the sample OD values to the standard curve.

### 2.4. Measurement of PT and PTT

#### 2.4.1. Prothrombin Time (PT) Test

Quantitative thromboplastin activity in rat plasma was measured using a PT determination kit (BioMed-Liquiplastin Diagnostics, Suzhou, China). Upon the addition of liquiplastin reagent to normal citrated plasma, the clotting mechanism was initiated, resulting in the formation of a solid gel clot at 37 °C within a specified time. The time taken for clot formation was recorded in seconds.

#### 2.4.2. Partial Thromboplastin Time (PTT) Test

PTT values were measured using an active partial thromboplastin time (APTT) kit (BIOLBO, Maizy, France). The reagent in the kit facilitates the recalcification of plasma in the presence of a standardized amount of cephalin (a platelet substitute) and a factor XII activator (kaolin) at 37 °C. This method minimizes reading time and optimizes optical detection.

### 2.5. Statistical Analysis

Results are expressed as mean ± standard error of the mean (if not otherwise stated). Differences between more than two groups were determined by the Kruskal–Wallis test and between two groups by the Mann–Whitney U test. The differences were statistically significant at *p* < 0.05. All statistical analyses were performed using IBM SPSS Statistics, version 27.

## 3. Results

Following mild and severe TBI, coagulation and fibrinolytic parameters in the rat model exhibited dynamic changes depending on the severity of the injury and the time points assessed post-impact. PT, PTT, D-dimer, and fibrinogen levels were measured at five time intervals, beginning 24 h post-injury and continuing through to 4 weeks. A total of 5 animals in the severe TBI group died within the first 24 h of the post-injury period, with no additional mortality observed during the subsequent 4-week follow-up period.

### 3.1. PT Response Following Mild and Severe TBI

**Figure 2:**
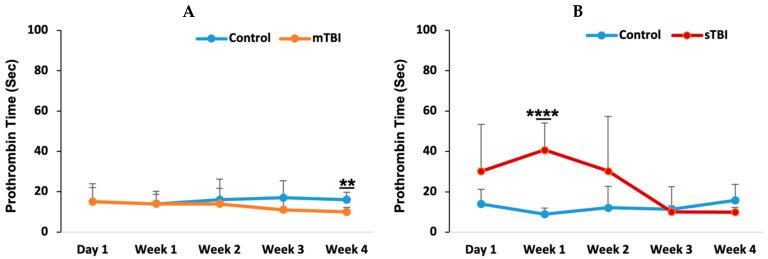
Prothrombin time (PT) following mild and severe TBI. Following mild TBI (**A**) and severe TBI (**B**), PT was measured at five time points: day 1, week 1, week 2, week 3, and week 4 post-injury. mTBI: mild TBI; sTBI: severe TBI. ** *p* < 0.01 and **** *p* < 0.0001.

Prothrombin time (PT) was assessed at five different time intervals: day 1, week 1, week 2, week 3, and week 4 post-injury. In the mild TBI group (Figure 2A), there were no significant changes in PT levels across the time intervals, except for a slight but statistically significant decrease at week 4 (*p* = 0.005). However, all PT values for both the control and mild TBI groups remained within the normal range (13.9–21.1 s) [41]. In contrast, the severe TBI group (Figure 2B) exhibited marked fluctuations in PT, with a significant increase observed at week 1 (*p* = 0.00015) compared to the control group. PT levels returned to baseline by week 3 and remained comparable to controls at week 4.

### 3.2. Partial Thromboplastin Time (PTT) Change in Mild and Severe TBI

**Figure 3:**
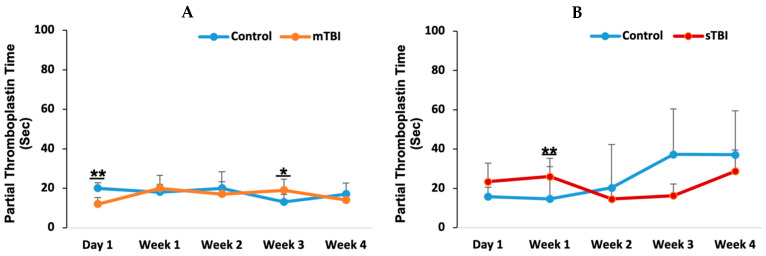
Partial thromboplastin time (PTT) following mild and severe TBI. Following mild TBI (**A**) and severe TBI (**B**), PTT was assessed at five time points: day 1, week 1, week 2, week 3, and week 4 post-injury. mTBI: mild TBI; sTBI: severe TBI. * *p* < 0.05 and ** *p* < 0.01.

To further assess the impact of TBI severity on coagulation function, partial thromboplastin time (PTT) was measured at five time points: day 1, week 1, week 2, week 3, and week 4 post-injury. In the mild TBI group (Figure 3A), PTT levels exhibited a transient decrease on day 1 compared to controls (*p* = 0.002). However, PTT levels subsequently returned to baseline within the first week post-injury. In contrast, the severe TBI group (Figure 3B) demonstrated a significant elevation in PTT (*p* = 0.01). Despite statistical significance, all PTT values across both mild and severe TBI groups remained within the normal reference range (15.2–33.9 s) [41].

### 3.3. D-Dimer Levels and Fibrinolytic Activity Following Mild and Severe TBI

**Figure 4:**
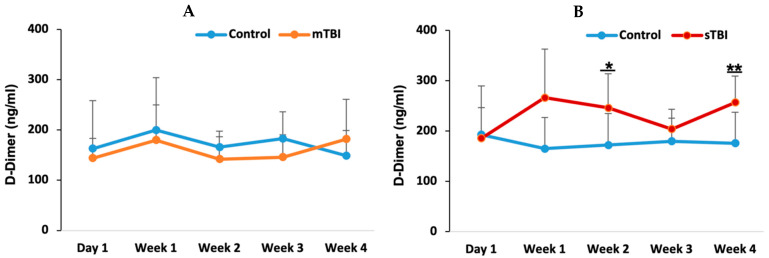
D-dimer following mild and severe TBI. D-dimer was measured at five time points following mild TBI (**A**) and severe TBI (**B**). mTBI: mild TBI; sTBI: severe TBI. * *p* < 0.05 and ** *p* < 0.01.

D-dimer levels were measured to evaluate coagulation and fibrinolytic activity at five time points: day 1, week 1, week 2, week 3, and week 4 post-TBI. In the mild TBI group (Figure 4A), there were no significant differences in D-dimer levels between the control and mild TBI groups at any time point, with values remaining within the normal range (3–200 ng/mL). This suggests that mild TBI may not have significantly impacted fibrinolytic activity.

In contrast, the severe TBI group (Figure 4B) showed a significant increase in D-dimer levels at week 2 (*p* = 0.024) and week 4 (*p* = 0.014) post-injury compared to the control group, with values exceeding the upper limit of the normal range. These elevations suggest increased fibrinolytic activity in response to the more severe injury.

### 3.4. Fibrinogen Concentrations After Mild and Severe TBI

**Figure 5:**
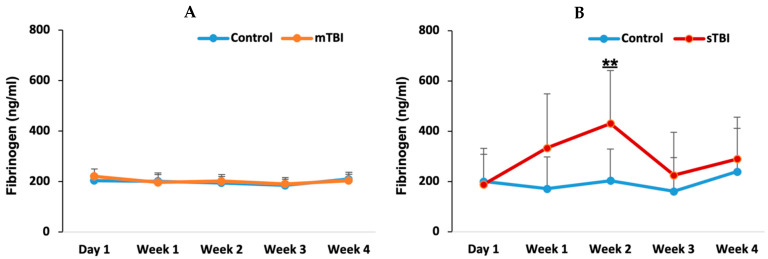
Fibrinogen levels following mild and severe TBI. Fibrinogen levels in mild TBI (mTBI) and severe TBI (sTBI). ** *p* < 0.01.

Fibrinogen levels were measured at five time points, similar to the previously mentioned test. In the mild TBI group (Figure 5A), no significant changes in fibrinogen levels were observed across the time points, and fibrinogen levels remained comparable to the control group throughout the study period. In contrast, the severe TBI group (Figure 5B) demonstrated a significant increase at week 2, with fibrinogen levels significantly higher than those in the control group (*p* = 0.011). This increase peaked at week 2, after which fibrinogen levels gradually decreased but remained elevated compared to the control group, though the differences were not statistically significant at weeks 3 and 4 (*p* = 0.54 and *p* = 0.38, respectively). Despite the marked increase in fibrinogen levels following severe TBI (Figure 5B), all fibrinogen values remained within the normal reference range (10–800 ng/mL).

## 4. Discussion

This study employs a 4-week longitudinal observation period, providing a critical contrast to the existing literature, which typically focuses on the first 24–72 h post-injury. Early diagnosis of patients with TBI is critical for effective management, guiding appropriate treatment, and preventing further complications related to hemodynamic coagulopathy. Assessing coagulation and fibrinolytic parameters such as PT, PTT, D-dimer, and fibrinogen as part of routine follow-up during the acute phase of mild and severe TBI provides valuable insights into the patient’s condition [20,31,42] and enables early intervention to improve outcomes in TBI patients. These parameters are key predictors of coagulopathy and abnormal fibrinolysis following TBI [25,35,36,38]. In this study, following both mild and severe TBI, the coagulation and fibrinolytic parameters, particularly in the severe model, demonstrated distinct hemostatic responses to varying injury severities. These findings reflect the dynamic and multifaceted nature of trauma-induced coagulopathy. The absence of platelet count and functional assessment is considered a limitation of this study, as platelets are an important component of the hemostatic system. Future studies incorporating platelet-related parameters may provide a more comprehensive evaluation of trauma-induced coagulopathy.

Following mild TBI, the unchanged PT values across different post-injury time points suggest that mild TBI may not significantly disrupt brain vessel integrity. The maintenance of PT values within the normal range further indicates that mild TBI does not strongly activate the coagulation cascade [43] or that any activation resolves quickly, as evidenced by the rapid formation and clearance of transient microthrombi within an hour post-injury [11]. The slight decrease in PT observed during the 4th week could represent a compensatory mild hypercoagulability state. However, despite statistical significance at certain time points, the decrease was transient, and the values remained within the normal physiological range. Therefore, this finding is unlikely to represent a clinically meaningful alteration in coagulation status and may instead reflect minor physiological fluctuations rather than a sustained hypercoagulable state.

In contrast, following severe TBI, PT increased significantly by week 1, exceeding normal levels. Previous studies have similarly reported PT elevations after severe TBI, with some showing an increase at the time of admission that persists for 3 h before normalizing [44]. Other studies indicate that PT peaks within 1–6 h post-injury and returns to normal within 12–18 h [45]. In our study, elevated PT was apparent from day 1 post-severe TBI compared to controls, with a significant peak observed during week 1. Severe TBI typically induces a strong coagulatory and inflammatory response, often leading to trauma-induced coagulopathy [44,46,47,48]. The elevated PT in week 1 likely reflects activation of the extrinsic coagulation pathway, potentially triggered by endothelial damage and blood–brain barrier disruption following TBI, which enhances tissue factor release [14,16,26,49]. Restoration of the BBB integrity may take several days or longer. Severe TBI is also a major cause of brain hemorrhage and intracranial bleeding [17,18,20,38,50], and this transient coagulopathy could result from the consumption of clotting factors in response to multiple brain vessel hemorrhages, leading to prolonged clotting times [24,25].

The fact that all PTT values following mild TBI remained within normal ranges suggests that the coagulation system of the body remained generally intact and responsive, avoiding a shift into a coagulopathic state. PTT values consistently stayed below 40 s [31] and, in another study, below 35 s [39]. The transient, slight changes in PTT observed on day 1 and week 3 post-injury may reflect the body’s immediate response to transient clot formation, indicating temporary shifts in the coagulation cascade as it manages tissue damage from mild TBI [11,31]. In contrast, following severe TBI, PTT increased in week 1, though it remained within the normal limits. However, patients with PTT values in the high normal range have been associated with a poor prognosis [44]. This rise in PTT suggests that not only was the extrinsic pathway (as indicated by PT) affected, but the intrinsic coagulation pathway was also activated and depleted by severe TBI [47]. Previous studies have shown that patients with abnormal PTT values experience progression of intracranial hemorrhage and have a higher risk of mortality [39], with PTT also being predictive of worse outcomes [31,45,51]. This could be due to widespread vascular injury and endothelial dysfunction, leading to the consumption of clotting factors in the early post-injury phase, particularly as PTT elevation coincides with increased PT.

D-dimer and fibrinogen levels showed no significant changes after mild TBI across all subgroups. D-dimer, a marker of fibrinolysis [23], remained stable, suggesting that mild TBI did not lead to substantial fibrin clot breakdown or a notable increase in thrombus formation. This stability supports the hypothesis that mild TBI induces only a limited systemic coagulation response, without triggering significant hemostatic changes related to fibrinolysis. Although there are limited studies on D-dimer levels following mild TBI, elevated D-dimer has been reported as a predictor of structural brain injury in some cases [21,22].

Fibrinogen, a key factor in clot formation, often remains stable or is not significantly depleted in less severe injuries, indicating that mild TBI may not cause substantial disruptions in the coagulation pathway or provoke pronounced hemorrhage. The absence of changes in fibrinogen levels further underscores the notion that mild TBI elicits a mild coagulatory response without significant involvement of the fibrinolytic system.

Following severe TBI, D-dimer levels were elevated in weeks 2 and 4, exceeding the normal range. Elevated D-dimer is a marker of both fibrinolytic and coagulation system activation [52], indicating substantial clot formation and subsequent breakdown. This finding reflects a hypercoagulable state followed by secondary fibrinolysis, a common occurrence in severe trauma [46,47]. Similar elevations in D-dimer levels have been reported in cases of severe TBI [44], aneurysmal subarachnoid hemorrhage [34], stroke [35], and disseminated intravascular coagulation [23] and have been associated with poor prognosis [53]. The delayed rise in D-dimer may suggest ongoing or resolving clot formation in response to vascular damage, and it has also been linked to an increased mortality rate [38]. Notably, D-dimer has been identified as a significant indicator of structural brain injury [22] and is considered the best coagulation/fibrinolytic parameter for predicting outcomes following TBI [44].

Fibrinogen levels were elevated during week 2 following severe TBI, aligning with the rise in D-dimer, but remained within the normal range. As a key component in clot formation, fibrinogen’s elevation suggests the body’s effort to mitigate bleeding and promote tissue repair. The return of fibrinogen to baseline levels after week 2 may reflect the resolution of the acute phase of trauma-induced coagulopathy, as the body stabilizes its hemostatic processes.

In our study, we investigated changes in coagulation and fibrinolytic parameters following varying severities of traumatic brain injury, including mild and severe cases, over a 4-week period divided into five time intervals. Previous studies have typically measured these parameters only for up to 72 h, suggesting that trauma coagulopathy peaks within the first 24 h [31]. Coagulopathy after TBI can manifest as either hypercoagulable or hypocoagulable states, potentially leading to secondary injuries through the induction of microthrombosis or the progression of hemorrhagic brain lesions [47].

Our findings indicate that there was an increase in coagulation parameters, including PT and PTT, during week 1. This may suggest the consumption of clotting factors to manage intracranial hemorrhage and injury to brain vessels following severe TBI, leading to prolonged clotting times [24,25]. Notably, during the first day following severe TBI, five animals in our study died, indicating a critical period within the first 24 h post-injury [18,39,54,55]. Although some mortality is expected following severe TBI, the relatively small number of deaths may still have introduced a degree of survival bias, as only surviving animals were included in the longitudinal analysis. From week 1 to week 2, coagulation parameters began to recover towards normal ranges, suggesting that the body was able to manage the coagulopathy status. Additionally, the breakdown of formed clots and the peak of the fibrinolytic process appeared to occur in week 2. Thus, our results highlight that coagulation and fibrinolytic parameters undergo dynamic changes in response to severe TBI [44,48].

## 5. Limitations

Despite the strengths of this longitudinal study, several limitations are noted. While the four-week observation period extends beyond typical acute studies, it may not capture the full spectrum of chronic, long-term hemostatic sequelae. In addition, the discrete time points sampled might have missed transient daily fluctuations in parameters.

## Data Availability

The raw data supporting the conclusions of this article will be made available by the authors on request.

## Abbreviations

TBI: traumatic brain injury; PT: prothrombin time; PTT: partial thromboplastin time; BBB: blood–brain barrier.
